# COmmunity and Single Microbe Optimisation System (COSMOS)

**DOI:** 10.1038/s41540-025-00534-w

**Published:** 2025-05-21

**Authors:** Lavanya Raajaraam, Karthik Raman

**Affiliations:** 1 Department of Biotechnology, Bhupat and Jyoti Mehta School of Biosciences, Indian Institute of Technology (IIT) Madras, Chennai, India; 2 Centre for Integrative Biology and Systems mEdicine (IBSE), Wadhwani School of Data Science and AI, IIT Madras, Chennai, India; 3 Department of Data Science and AI, Wadhwani School of Data Science and AI, IIT Madras, Chennai, India

**Keywords:** Biotechnology, Computational biology and bioinformatics, Systems biology

## Abstract

Bioprocessing utilises microbial monocultures and communities to convert renewable resources into valuable products. While monocultures offer simplicity, communities provide metabolic diversity and cooperative biosynthesis. To systematically evaluate these systems, we developed COmmunity and Single Microbe Optimisation System (COSMOS), a dynamic computational framework that simulates and compares monocultures and co-cultures to determine optimal microbial systems tailored to a specific environment. COSMOS revealed key factors shaping biosynthetic performance, such as environmental conditions, microbial interactions, and carbon sources. Notably, it predicted the *Shewanella oneidensis*–*Klebsiella pneumoniae* co-culture as the most efficient producer of 1,3-propanediol under anaerobic conditions, aligning closely with experimental data, including optimal carbon source concentrations and inoculum ratios. Additional findings highlight the resilience of microbial communities in nutrient-limited processes and emphasise the role of computational tools in balancing productivity with operational simplicity. Overall, this study advances the rational design of microbial systems, paving the way for sustainable bioprocesses and circular bio-economies.

## Introduction

Biomanufacturing supports sustainable development by converting renewable resources, such as agricultural waste or wastewater, into valuable products like biofuels, pharmaceuticals, and bioplastics^1^. Both microbial monocultures and communities are used in these processes, each with distinct strengths and challenges. Monocultures are easier to control, manipulate, and engineer for specific product yields, making them ideal for simple, well-characterised bioprocesses. However, their productivity can reach a plateau, even after genetic optimisation^2^.

Microbial communities, on the other hand, leverage complementary metabolic capabilities and interspecies cooperation^3^. This has sparked significant interest in engineering co-cultures tailored to achieve specific bioprocess objectives^4^. Studies have highlighted the advantages of co-cultures over monocultures in enhancing biosynthetic efficiency. A molecular toolkit of auxotrophic and overexpression yeast strains was developed, enabling the construction of diverse two- and three-member communities with distinct metabolic capabilities^5^. Co-cultures often exhibit superior biosynthetic potential compared to their constituent monocultures. For instance, *Clostridium thermocellum* and *Clostridium thermosaccharolyticum* co-cultures achieved a 94.1% higher yield of hydrogen than their monocultures^6^, while *Clostridium thermocellum* and *Clostridium thermolacticum* co-cultures demonstrated up to a twofold increase in ethanol yield^7^.

Similarly, heterologous communities frequently outperform monocultures due to enhanced metabolic cooperation and resource sharing. For example, a co-culture of *Escherichia coli* and *Saccharomyces cerevisiae* produced high amounts of taxanes, whereas neither monoculture generated detectable levels^8^. These advantages extend to complex substrate utilisation, where certain organisms degrade substrates like lignin or cellulose into simpler metabolites that can be further metabolised by other community members, facilitating resource-sharing^9,10^. Communities are also less prone to feedback inhibition because one species may utilise by-products of another, leading to enhanced growth and stability^11^. Furthermore, the ‘division of labour’ across species helps distribute the metabolic burden, improving production efficiency for complex products^12^. However, communities are inherently harder to manage and require careful optimisation to maintain stability and maximise productivity.

Given these trade-offs, the choice between monocultures and communities is dependent on the nature of the bioprocess. The cooperative behaviour of communities provides resilience and efficiency^13^, whereas monocultures are well-studied and can be easier to manipulate. Designing effective bioprocesses involves identifying the most suitable microbial system for specific substrates or desired products. The decision is not always as straightforward as selecting communities for lignocellulosic biomass conversion and monocultures for simpler fermentations^14^. Selecting the optimal microbial system for other processes can be challenging, as it would require testing numerous combinations of organisms and environmental conditions—an approach that is both labour-intensive and impractical^15^. Therefore, computational algorithms become essential for managing this complexity and streamlining the selection process^16^.

While some algorithms optimise specific co-cultures, few can systematically evaluate multiple microbial systems to identify the most suitable option for a given environment. For instance, an algorithm has been developed to optimise substrate-pulsing to obtain stable communities^17^. Other studies have explored strategies to induce microbial communities for specialised product synthesis, with various computational approaches developed to regulate these processes^18^. Additionally, recent efforts^19^ have focused on modelling growth kinetics in single co-cultures, revealing how interspecies interactions, such as cell fusion, can be harnessed to enhance bioproduction.

Other algorithms like FLYCOP^20^ optimise parameters such as inoculation ratio and timing for a single microbial consortium, making it useful for fine-tuning a predefined community for a specific bioprocess. However, it does not facilitate the comparative analysis of multiple communities to identify the most effective configuration. Yet another study^21^ sought to optimise co-cultures by identifying suitable bacterial partners for anaerobic fungi. However, their approach lacks a systematic framework that can be directly applied to different microbial systems and does not incorporate a comparative analysis of monocultures. Evaluating co-cultures in isolation may overlook cases where monocultures provide superior performance, highlighting the need for a more comprehensive assessment.

To address these limitations, we introduce COmmunity and Single Microbe Optimisation System (COSMOS), a computational framework that systematically compares both communities and monocultures to determine the optimal microbial system for a given bioproduct. While COSMOS can simulate three-member and larger communities, the combinatorial explosion of possibilities makes it intractable to exhaustively evaluate all the combinations. Therefore, this study primarily focuses on co-cultures. Using COSMOS, we construct pairwise communities from a predefined set of organisms and apply dynamic modelling to evaluate their performance. COSMOS integrates dynamic Flux Balance Analysis (FBA)^22,23^ and Flux Variability Analysis (FVA) to simulate the growth of communities and their constituent monocultures^24^. Unlike some studies that assess community performance relative to the average productivity of its monocultures, COSMOS benchmarks each community against the highest-performing monoculture^6^. This approach ensures that the optimal microbial system is identified based on the highest yield/productivity rather than simply determining whether the community outperforms the sum of its parts. It also enables users to make informed decisions based not only on productivity or yield but also on additional factors, such as community abundance distribution.

In summary, this work contributes to the development of sustainable biomanufacturing by identifying when communities or monocultures should be used and how bioresources can be efficiently employed. It marks a step forward in creating economically viable, eco-friendly processes, aligning with the global effort to promote sustainable industrial practices. This framework aligns with the growing need for optimised bio-based processes that reduce waste and reliance on fossil resources, promoting a circular economy.

## Results

### Navigating trade-offs: choosing between monocultures and communities

The medium composition is one of the most substantial factors that can affect a bioprocess. We come across both nutrient-dense or ‘rich’ feedstock, like animal manure and sludge, which have more nitrogen content and trace minerals, and ‘less-dense’ feedstock, like wheat straw and corn stover^25^, which may lack essential micronutrients. The biosynthetic capability of the organisms can also be affected as a result of this medium composition^26^. So, we need to either choose the target product based on the feedstock availability or source the best-suited feedstock for our product of interest. Using COSMOS, we analyse the effect of medium composition on the biosynthetic capability of a diverse set of organisms.

**Fig. 1:**
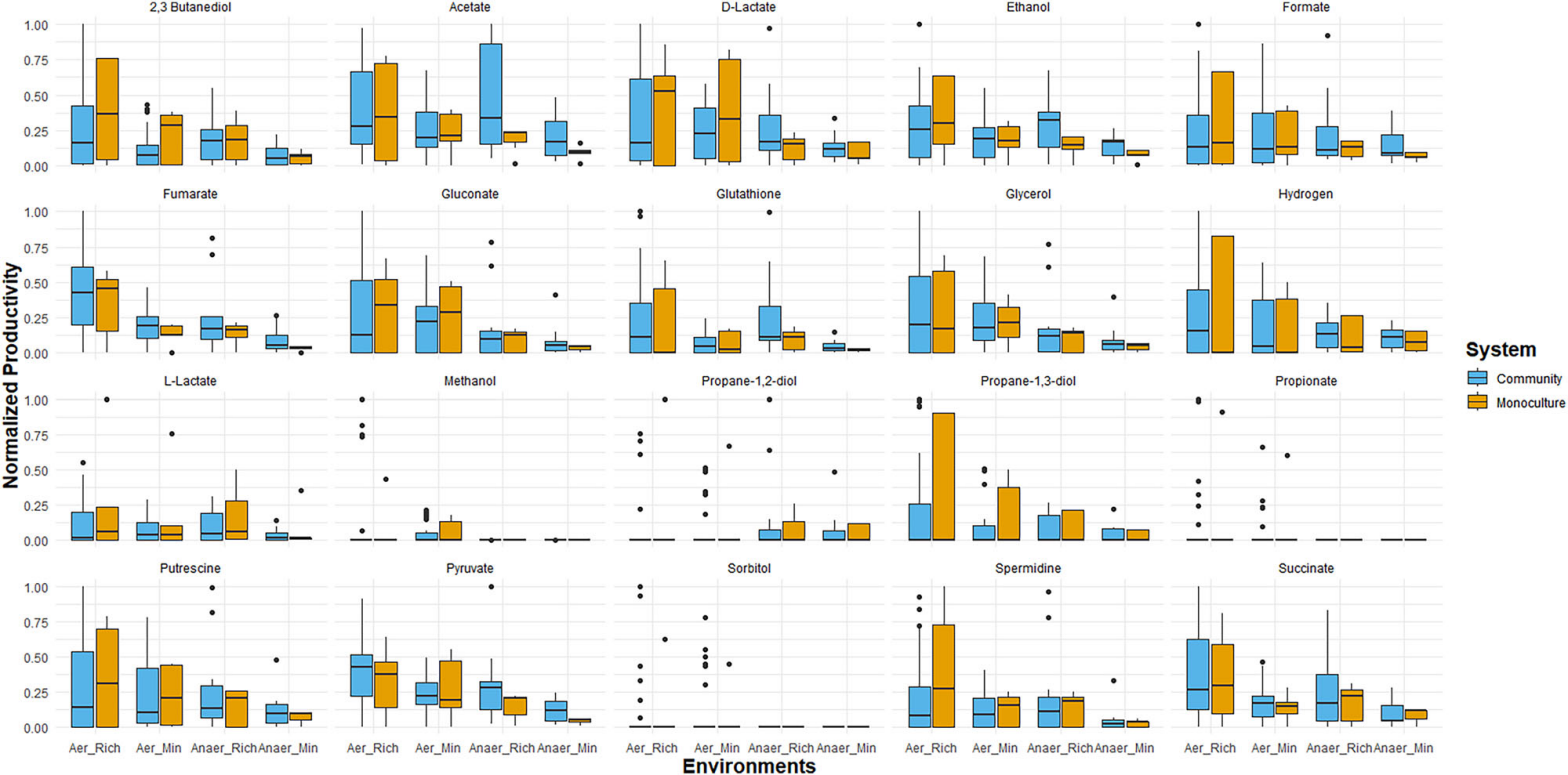
Productivity of communities and monocultures across varying environmental conditions. The productivity of both communities and monocultures for products across all four environments is represented. Five products, viz. adipic acid, xylitol, catechol, butanol and butyrate, were excluded as they are not produced in any of the organisms under study.

To examine the impact of medium composition, simulations were conducted as outlined in Section 4.3. Pairwise co-cultures (^9^C_2_) were assessed under four distinct environmental conditions: aerobic-rich, aerobic-minimal, anaerobic-rich, and anaerobic-minimal. We calculated the productivity of each product for both communities and the constituent monocultures in all four environments, as shown in Fig. 1.

When comparing the four environments, we found that overall productivity is highest in the aerobic-rich environment (Fig. 1). However, we found the effect of the environment on microbial systems to be more nuanced and product-dependent. While monocultures, on average, performed better in the aerobic-rich environment, communities often achieved the highest productivity for specific products. The list of microbial systems with maximum productivity for the aerobic-rich environment is summarised in Table 1. Our results highlight the importance of conducting an analysis tailored to the specific product and environmental conditions to identify the optimal microbial system.

**Table 1:** Optimal microbial for four products in the aerobic-rich environment

#	Fumarate			Ethanol			Spermidine			Glutathione		
	Microbial system	Productivity (mmol/L/h)	Abundance	Microbial system	Productivity (mmol/L/h)	Abundance	Microbial system	Productivity (mmol/L/h)	Abundance	Microbial system	Productivity (mmol/L/h)	Abundance
1	*P. aeruginosa - K. pneumoniae*	1.28	0.85, 0.15	*S. oneidensis - K. pneumoniae*	2.04	0.19, 0.81	*E. coli*	0.07	1	*S. oneidensis - K. pneumoniae*	0.26	0.19, 0.81
2	*E. coli - K. pneumoniae*	1.22	0.60, 0.40	*S. cerevisiae - K. pneumoniae*	1.42	0.13, 0.87	*B. subtilis*	0.05	1	*E. coli - K. pneumoniae*	0.25	0.60, 0.40
3	*S. oneidensis - K. pneumoniae*	1.10	0.19, 0.81	*K. pneumoniae*	1.29	1	*S. cerevisiae - L. lactis*	0.03	0.84,0.16	*E. coli - P. putida*	0.19	0.72, 0.28
4	*E. coli - P. putida*	1.07	0.72, 0.28	*E. coli*	1.10	1	*S. cerevisiae*	0.02	1	*E. coli*	0.17	1
5	*B. subtilis - K. pneumoniae*	0.88	0.15, 0.85	*P. aeruginosa - S. oneidensis*	0.63	0.89, 0.11				*B. subtilis - K. pneumoniae*	0.13	0.15, 0.85

**Fig. 2:**
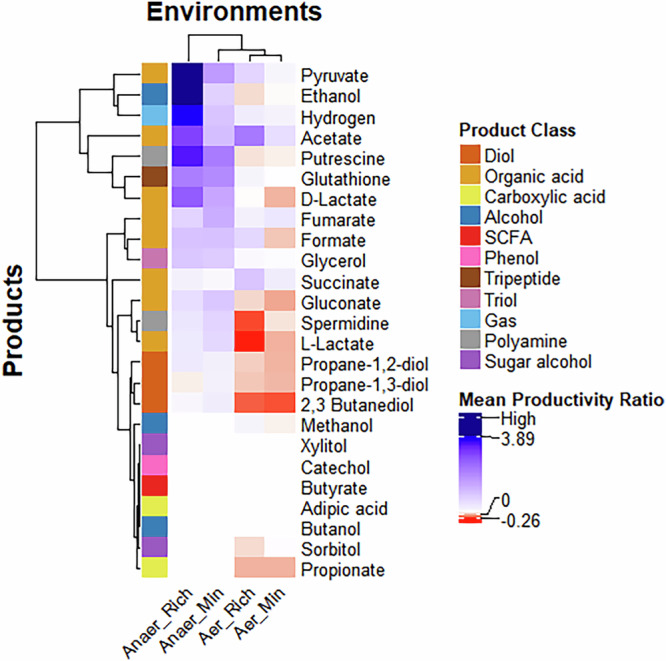
Effect of medium composition and oxygen availability on the productivity of communities vs monocultures. The average productivity ratio of communities across all products and environments is represented. Positive values (blue) indicate increased productivity in communities compared to monocultures, while negative values (red) indicate a decline. In cases where monoculture productivity is extremely low, leading to an exceptionally high productivity ratio, we classify it as ‘High’ and denote it with a navy-blue colour. Hierarchical clustering was conducted using the complete linkage method.

To efficiently compare the productivity of communities and monocultures, we calculated the productivity ratio for all products across the four environments. It should be noted that the maximum productivity of the two monocultures was compared against the productivity of the co-culture. The productivity ratios across all four environments are represented in Fig. 2.

In a co-culture with organisms A and B, where

1 $${{Mono}}_{A}+{{Mono}}_{B}={{Comm}}_{{AB}}$$

the change in productivity is given as

2 $$\Delta {Productivity}={Productivity}\left({{Comm}}_{{AB}}\right)-\max \left({Productivity}\left({{Mono}}_{A}\right),{Productivity}\left({{Mono}}_{B}\right)\right)$$

and the ratio of productivity of the co-culture to that of the monoculture is given as

3 $${Productivity\; ratio}=\frac{\Delta {Productivity}}{{abs}\left(\max \left({Productivity}\left({{Mono}}_{A}\right),{Productivity}\left({{Mono}}_{B}\right)\right)\right)}$$

When comparing the productivity ratio of communities and monocultures, we find that the anaerobic-rich environment predominantly favours community-based production, while monocultures perform best in the aerobic-minimal medium ($p=2.38\times {10}^{-10}$; Supplementary Data 5). One possible explanation is that the incomplete nature of anaerobic fermentation can lead to lower productivity in monocultures. In contrast, microbial communities may exchange primary metabolites, enhancing both growth and productivity, particularly in anaerobic conditions. To test this hypothesis, we analysed the abundance ratios of five communities across all four environmental conditions, as shown in Table 2. Under anaerobic conditions, although the total biomass decreases, the growth rates of both organisms become more balanced, leading to a more stable abundance ratio. This equilibrium likely contributes to the improved performance of microbial communities in anaerobic environments, often resulting in more positive or mutualistic interactions, as discussed in Section 2.2. Additionally, previous studies have shown that increased metabolite exchange and cooperative interactions can lead to higher productivity in microbial communities^27^.

**Table 2:** Comparison of microbial communities exhibiting growth across all environments

	Aerobic-rich				Aerobic-minimal				Anaerobic-rich				Anaerobic-minimal			
Co-culture	Biomass	Total biomass	Abundance	Interaction	Biomass	Total biomass	Abundance	Interaction	Biomass	Total biomass	Abundance	Interaction	Biomass	Total biomass	Abundance	Interaction
*E. coli - L. lactis*	0.76, 0.17	0.93	0.82, 0.18	Commensalism	0.57, 0.13	0.7	0.81, 0.19	Parasitism	0.35, 0.23	0.58	0.60, 0.40	Mutualism	0.19,0.24	0.43	0.44, 0.56	Mutualism
*L. lactis - S. oneidensis*	0.13, 0.35	0.48	0.26, 0.74	Parasitism	0.13, 0.36	0.49	0.26, 0.74	Parasitism	0.14, 0.22	0.36	0.38, 0.62	Parasitism	0.13,0.22	0.35	0.38, 0.62	Parasitism
*L. lactis - K. pneumoniae*	0.12, 0.80	0.92	0.13, 0.87	Parasitism	0.12, 0.35	0.47	0.15, 0.85	Parasitism	0.13, 0.48	0.61	0.21, 0.79	Parasitism	0.14,0.29	0.43	0.32, 0.68	Parasitism
*B. subtilis - K. pneumoniae*	0.14, 0.80	0.94	0.15, 0.85	Parasitism	0.14, 0.66	0.8	0.18, 0.82	Parasitism	0.17, 0.46	0.63	0.27, 0.73	Parasitism	0.17,0.29	0.46	0.37, 0.63	Commensalism
*S. oneidensis - K. pneumoniae*	0.19, 0.78	0.97	0.19, 0.80	Commensalism	0.15, 0.64	0.79	0.19, 0.81	Parasitism	0.23, 0.48	0.71	0.32, 0.68	Mutualism	0.25,0.27	0.52	0.47, 0.53	Mutualism

To further investigate the role of metabolite exchange and cross-feeding in this shift, we analysed the exchanged metabolites in the *S. oneidensis—K. pneumoniae* co-culture across all four environments. As shown in Table 3, cross-feeding was more pronounced in the anaerobic minimal environment compared to the aerobic-rich environment. While the aerobic-minimal and anaerobic-minimal environments facilitated the exchange of similar metabolites, their quantitative effects differed—leading to a parasitic interaction in the aerobic-minimal environment, which shifted to mutualism in anaerobic conditions. Since metabolite concentrations fluctuate over time, we have not included absolute concentration values. The observed equalisation of growth rates and increased cross-feeding under anaerobic conditions provide insights into why microbial communities may exhibit improved performance in such environments.

**Table 3:** Cross-feeding in *S. oneidensis - K. pneumoniae* Co-culture across all environments

Aerobic-rich medium			Aerobic-minimal medium			Anaerobic-rich medium			Anaerobic-minimal medium		
Metabolite	*S. oneidensis*	*K. pneumoniae*	Metabolite	*S. oneidensis*	*K. pneumoniae*	Metabolite	*S. oneidensis*	*K. pneumoniae*	Metabolite	*S. oneidensis*	*K. pneumoniae*
Ethanol	Consumer	Producer	Acetate	Consumer	Producer	Ethanol	Consumer	Producer	Acetate	Consumer	Producer
Serine	Producer	Consumer	Ethanol	Consumer	Producer	Serine	Producer	Consumer	Ethanol	Consumer	Producer
H^+^ ions	Consumer	Producer	Formate	Consumer	Producer	Valine	Consumer	Producer	Formate	Consumer	Producer
			Fumarate	Consumer	Producer	H^+^ ions	Consumer	Consumer	Fumarate	Consumer	Producer
			H^+^ ions	Consumer	Producer				H^+^ ions	Consumer	Producer

While general trends suggest that co-cultures often have better productivity in anaerobic environments, notable exceptions exist where certain communities thrive in aerobic environments. For example, in the aerobic-rich medium, the *S. cerevisiae—Bacillus subtilis* (0.066 mmol/L/h) and *S. cerevisiae—Lactococcus lactis* (0.06 mmol/L/h) co-cultures exhibited the highest sorbitol productivity, representing a 59% and 48% increase, respectively, compared to the monocultures. *B. subtilis* and *L. lactis* did not produce excess sorbitol as monocultures, while *S. cerevisiae* alone achieved a productivity of 0.04 mmol/L/h. Therefore, in co-culture, although *B. subtilis* and *L. lactis* do not produce sorbitol, they enhance *S. cerevisiae*’s sorbitol production, likely through metabolic interactions and resource exchange. Likewise, in the anaerobic-minimal medium*, B. subtilis* outperforms all the other microbial communities in the production of L-lactate, with the productivity of *B. subtilis* being 0.29 mmol/L/h. It is to be noted that the second-best alternative is *E. coli—Pseudomonas aeruginosa* co-culture, with a productivity of 0.12 mmol/L/h of L-lactate, which is less than half of the productivity shown by *B. subtilis*. These examples illustrate that while general trends offer valuable insights, exceptions can be leveraged strategically to optimise bioprocesses. For instance, certain microbial communities thrive in aerobic environments, whereas some monocultures, such as *B. subtilis*, outperform communities in anaerobic minimal environments. If the differences in productivity are minimal, monocultures may be preferable due to easier process control, whereas co-cultures can be advantageous if they offer superior biosynthetic capabilities.

Therefore, a systematic screening of both monocultures and communities is essential. In the following sections, we explore the impact of additional factors, such as interaction type, carbon sources and initial biomass ratio, on productivity and community dynamics.

### Synergy in action: positive interactions drive higher productivity

As we are dealing with pairwise communities, another interesting factor that we can analyse is the effect of interaction. To find the interaction type of a co-culture, we compare the growth of the organism as a monoculture and in the community under the same environmental conditions. The communities were categorised into six interaction types—competition (−/−), amensalism (−/0), parasitism (+/−), neutralism (0/0), commensalism (+/0), and mutualism (+/+)—based on a ${threshold}$ of 10% difference in growth rates of the microbe in the co-culture and the monoculture^28^.

In a co-culture with organisms A and B,

interaction is positive (+) if

4 $${{Comm}}_{{A|B}} > \left(1+{threshold}\right)* \left(\frac{{{Mono}}_{{A|B}}}{2}\right)$$

interaction is negative (−) if

5 $${{Comm}}_{\left({A|B}\right)} < \left(1-{threshold}\right)* \left(\frac{{{Mono}}_{{A|B}}}{2}\right)$$

and interaction shows no change (0) if

6 $${\left(1-{threshold}\right)* \left(\frac{{{Mono}}_{{A|B}}}{2}\right) < {Comm}}_{{A|B}} < \left(1+{threshold}\right)* \left(\frac{{{Mono}}_{{A|B}}}{2}\right)$$

**Fig. 3:**
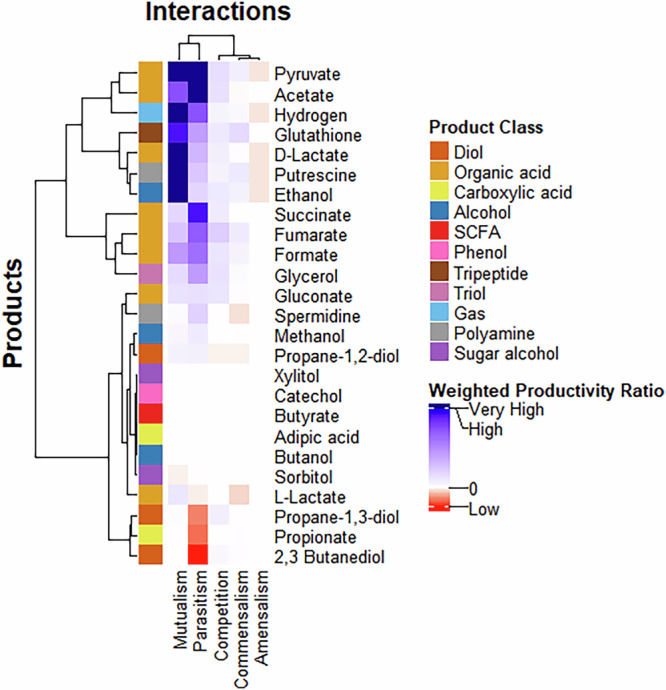
Effect of interaction type on the productivity of communities vs monocultures across products. Positive values (blue) indicate an increase in the productivity of a given product in the co-culture compared to the monocultures, while negative values (red) reflect a decline in productivity. Neutralism was not observed. Hierarchical clustering was conducted using the complete linkage method.

The average ${Productivity\; ratio}$ was calculated for each product across the four environments, as shown in Fig. 3. Mutualistic interactions generally lead to the highest productivity gains in communities, likely due to the increased biomass of the participating organisms. Parasitic interactions follow, with productivity varying depending on the product—some are better produced in communities, while others are more efficiently generated in monocultures. This variation may arise because, in some cases, the producer strain exhibits higher growth, whereas in others, it experiences reduced growth. The effect of interaction type on the productivity ratio was statistically significant ($p=1.4\times {10}^{-10}$; Supplementary Data 6). However, no significant differences were observed among competition, commensalism, and amensalism, suggesting that these interaction types have minimal impact on productivity differences between communities and monocultures. The influence of environmental conditions on interaction types can be further understood by examining Table 2. As conditions shift from aerobic to anaerobic, microbial interactions become increasingly cooperative, transitioning from negative to more positive. This can be attributed to the slower, less efficient nature of anaerobic fermentation, which equalises growth rates and promotes metabolite exchange between species, as evident from Tables 2 and 3. The resulting resource-sharing dynamics foster mutualistic relationships, highlighting the significant influence of oxygen availability on community interactions.

### Tailored metabolism: communities and monocultures show distinct carbon preferences

**Fig. 4:**
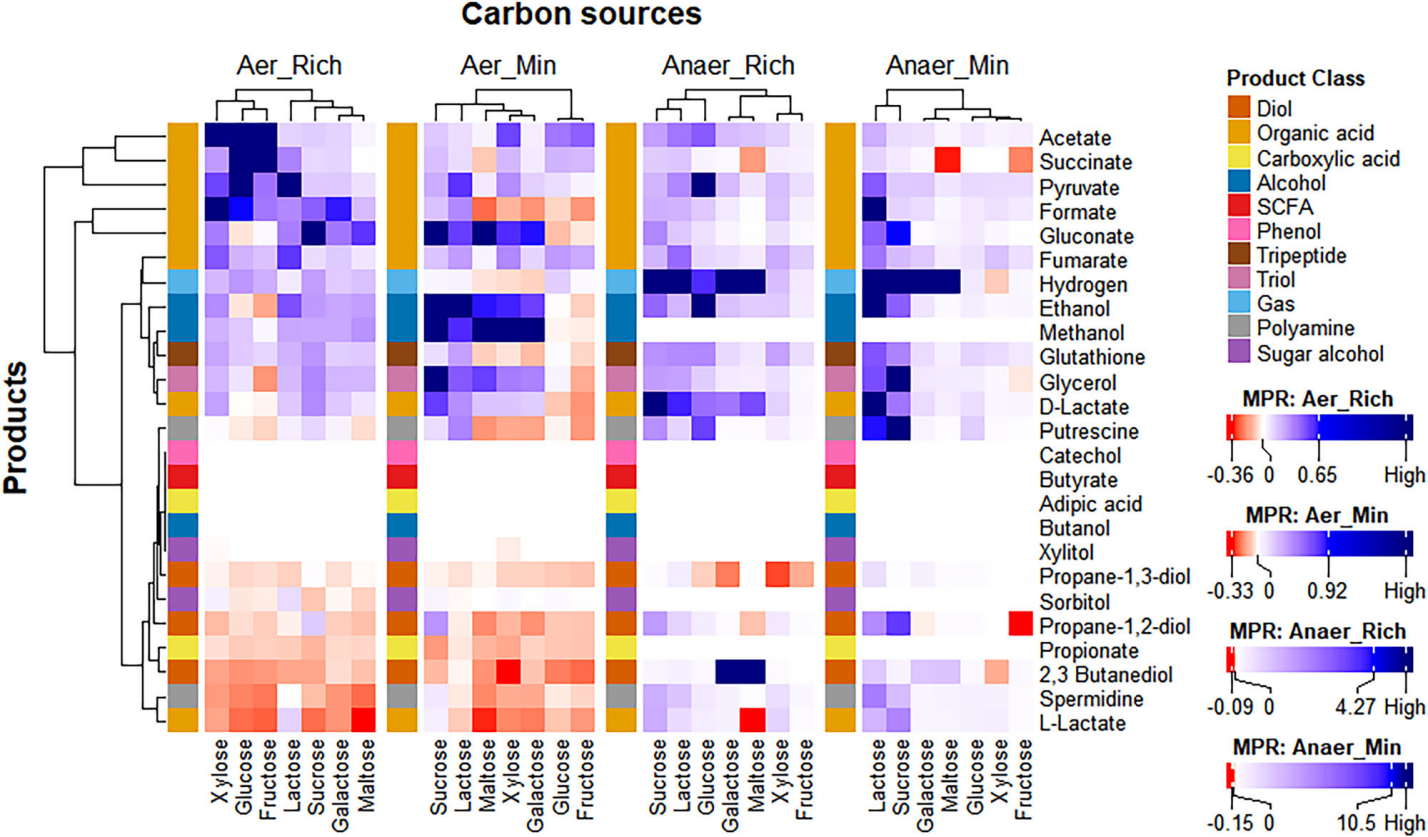
Effect of carbon source on the productivity of communities vs monocultures. The effect of carbon sources on the ${Productivity\; ratio}$ is compared across all four environments. Positive values (blue) indicate an increase in the productivity of a given product in the community compared to the monocultures, while negative values (red) reflect a decline in productivity. Hierarchical clustering was conducted using the complete linkage method.

Another key factor we examined is the impact of different carbon sources on the growth and biosynthetic capabilities of microbial systems. In this analysis, we replaced the glucose in the ‘rich’ and ‘minimal’ media with 10 mmol/L of the carbon source under investigation. We tested seven different carbon sources and compared the productivity of communities and monocultures across all four environments, as shown in Fig. 4.

Our findings show that while there are some variations, lactose and sucrose consistently enhance productivity in communities, whereas xylose and fructose favour monocultures. Interestingly, the aerobic-rich medium deviates from the pattern observed in the other three environments. While xylose and fructose promote higher productivity in communities in the aerobic-rich environment, these carbon sources support higher productivity in monocultures in other environments. Although the underlying cause remains unclear, this provides an additional factor that could be manipulated to optimise the bioprocess. This insight can guide the design or supplementation of fermentation media such that the choice of carbon source aligns with the microbial system and environment. The effect of carbon source on the productivity ratio was statistically significant ($p=1.8\times {10}^{-4}$; Supplementary Data 7).

To explore the variation amongst communities, we calculated the productivity ratio for four different products across communities in the aerobic-rich medium, as shown in Supplementary Fig. 1. Some communities, like *S. oneidensis—K. pneumoniae*, showed minimal sensitivity to various carbon sources. However, others, such as *S. cerevisiae—L. lactis*, displayed varying productivity across carbon sources. In some cases, like *E. coli—S. cerevisiae*, the community does not grow under specific carbon sources. Moreover, this behaviour can also be product-dependent. Thus, product-specific analysis is crucial for identifying the optimal combination of carbon source and microbial system.

### Computational screening reveals ideal microbial systems across fermentation conditions

While communities generally perform better in challenging environments, exceptions may occur. Therefore, it is essential to analyse the specific product of interest before selecting the most suitable microbial system. Furthermore, variations between communities mean that choosing the optimal community for a given nutrient source is crucial. Since experimentally comparing multiple communities is time-consuming and labour-intensive, computational analysis becomes essential.

**Fig. 5:**
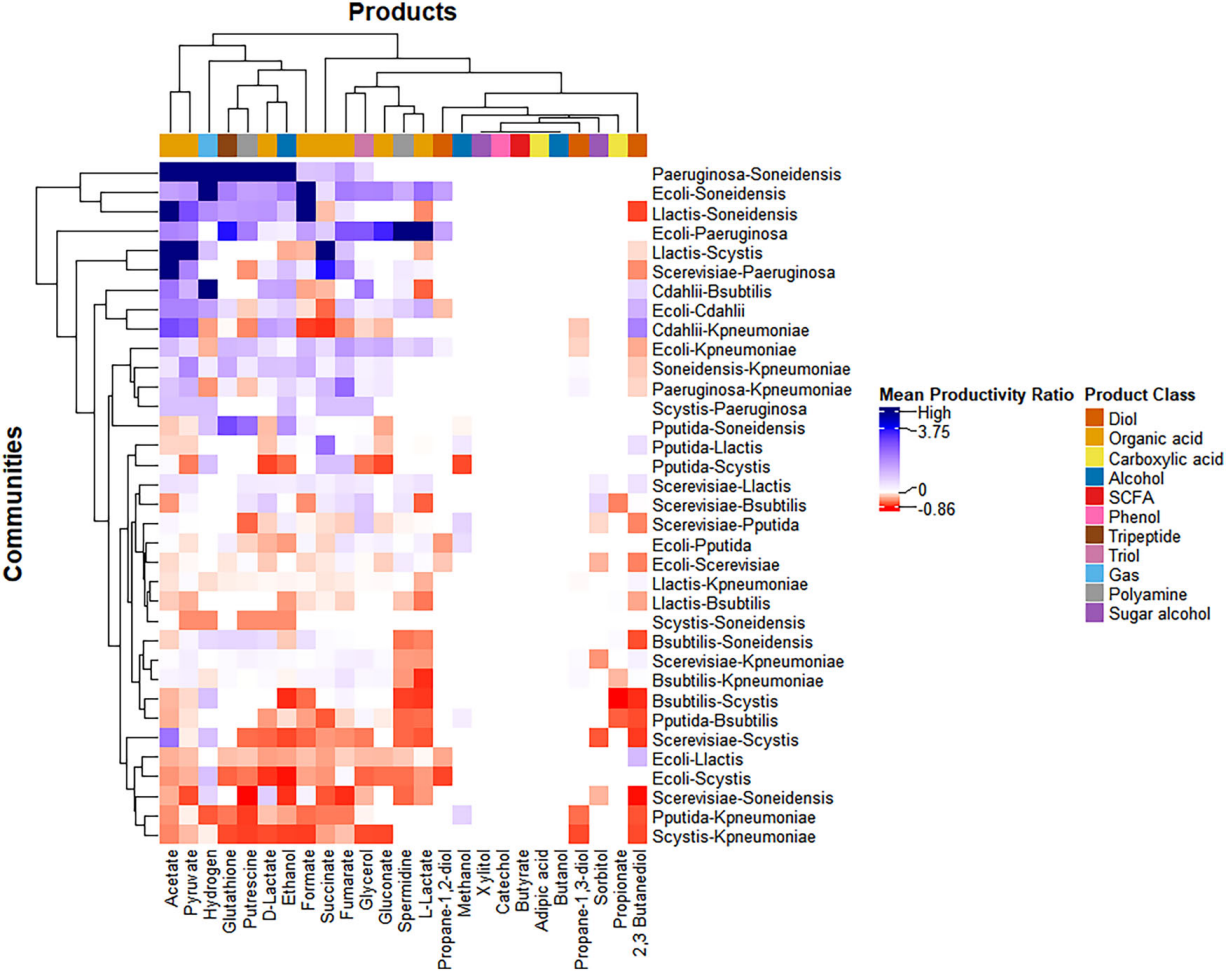
Comparison of Productivity of Communities and Monocultures. This figure presents the mean productivity ratio of communities, averaged across four environments, for all the products under study. Positive values (blue) indicate an increase in the productivity of a given product in the co-culture compared to the monocultures, while negative values (red) reflect a decline in productivity. Hierarchical clustering was conducted using the complete linkage method.

We compared the change in productivity between communities and monocultures across all products, as shown in Fig. 5. The mean productivity ratio for 25 products was calculated across the four environments for each community. Positive values (blue) indicate higher productivity in communities compared to their constituent monocultures, while negative values (red) indicate lower productivity. Clear clustering patterns emerge both by product and community, highlighting preferences in microbial systems. The effect of community on the productivity ratio was found to be statistically significant, with a ($p=2.2\times {10}^{-16}$; Supplementary Data 8).

Microbial communities such as *P. aeruginosa–S. oneidensis* and *E. coli–S. Oneidensis* generally outperforms their monocultures, particularly in the production of metabolites like pyruvate, hydrogen, and glutathione. This behaviour may be attributed to the proficiency of *S. oneidensis* in electron transfer, which enhances the metabolic activity of co-culture partners, thereby improving overall community performance^29^. In contrast, communities like *Synechocystis* spp.*–K. pneumoniae* and *P. putida–K. pneumoniae* consistently exhibit lower productivity compared to their respective monocultures. This could be due to inherent biological differences that hinder cooperation, resulting in limited biosynthetic capabilities. In such cases, monocultures may utilise available resources more efficiently without competition, leading to superior performance^27^. Furthermore, metabolite production appears to be highly product- and organism-dependent. For instance, acetate and pyruvate are more efficiently synthesised in co-culture systems, whereas sorbitol and propionate yield higher levels in monocultures. Additionally, productivity patterns vary across different environmental conditions, underscoring the importance of evaluating microbial systems in a context-specific manner.

To investigate this behaviour further, we identified the microbial systems with maximum productivity for producing four key products from different classes under all four environmental conditions, as summarised in Supplementary Table 5. The observations reveal that productivity is highest in the aerobic-rich environment, as discussed in Sections 2.1 and 2.2. Notably, the anaerobic-rich environment also performs competitively and, in some cases, surpasses the aerobic minimal medium in productivity. The findings in Supplementary Table 5 show that some of the best-performing systems in aerobic environments are monocultures, while communities tend to outcompete monocultures in anaerobic environments. It also highlights variability within products—while fumarate is consistently better produced by communities across all environments, spermidine is predominantly produced by monocultures in most conditions. This highlights the necessity of selecting the optimal microbial consortium and environmental setting tailored to the specific product of interest.

Moreover, when choosing a community, the selection criteria need not be restricted to productivity but can also be extended to other factors like abundance ratios. When two systems exhibit similar productivity, choosing the one with a more balanced abundance may be preferable, as it can lead to a more stable community. Moreover, the final choice can also be guided by biological insights, such as ease of handling or specific biological characteristics desirable for the bioprocess of interest. The optimal systems in the other environments are provided in Supplementary Data 1–4.

Additionally, we compiled the top-performing systems for all 25 products across four environments in Supplementary Table 6. As expected from previous analyses, monocultures dominate in the aerobic-rich medium. Meanwhile, communities excel in the anaerobic minimal medium, reinforcing the idea that harsher conditions favour biosynthesis in the communities. The detailed results for all 25 products are in Supplementary Data 1–4, with key findings aligning with experimental studies summarised in Table 4 alongside references.

**Table 4:** Results that align closely with experimental findings

#	Product	System name	Productivity in Co-culture (mmol/L/h)	Productivity in Monoculture A (mmol/L/h)	Productivity in Monoculture B (mmol/L/h)	Improvement in productivity (%)	Abundance	Environment	Reference
1	1,3 - Propanediol	*S. oneidensis - K. pneumoniae*	0.064	0	0.0519	23.31	0.32, 0.68	Anaerobic-rich	^29^
2		*P. aeruginosa - K. pneumoniae*	0.053	0	0.018	196.11	0.58, 0.42	Anaerobic-minimal	^71^
3	2,3 - Butanediol	*P. aeruginosa - K. pneumoniae*	0.073	0	0.036	103.06	0.58, 0.42	Anaerobic-minimal	^71^
4	Hydrogen	*C. ljungdahlii - B. subtilis*	0.684	0.091	0	647.92	0.87, 0.13	Anaerobic-rich	^72^
5	Ethanol	*S. cerevisiae – B. subtilis*	0.630	0.416	0.604	4.41	0.69, 0.31	Aerobic-rich	^73^
6		*S. cerevisiae - L. lactis*	0.584	0.416	0.009	40.22	0.84, 0.16	Aerobic-rich	^74^
7		*S. cerevisiae - L. lactis*	0.410	0.363	0.008	13.03	0.65, 0.35	Aerobic-minimal	^75^

### Optimising biomass ratios enhances productivity in microbial communities

The initial biomass ratio of organisms within a community can significantly influence growth, abundance, and overall community dynamics. By adjusting the inoculum ratio, we can further enhance the biosynthetic capabilities of the community. Tables 5 and 6 illustrate how variations in inoculum ratios impact 1,3-propanediol (1,3-PDO) production in *P. aeruginosa - K. pneumoniae* and *S. oneidensis - K. pneumoniae* communities. Product concentrations vary with inoculum ratios, highlighting distinct responses between the two communities. While increasing *K. pneumoniae* inoculum enhances product titre in both, the *S. oneidensis - K. pneumoniae* co-culture shows an increase in total biomass, whereas *P. aeruginosa - K. pneumoniae* displays a growth reduction. Moreover, higher *K. pneumoniae* inoculum in *S. oneidensis - K. pneumoniae* leads to a more skewed abundance ratio. These findings suggest that inoculum ratios can have varied effects on community dynamics and biosynthetic output, emphasising the need for careful ratio optimisation.

**Table 5:** Effect of inoculum ratio on the productivity of 1,3-PDO in *P. aeruginosa - K. pneumoniae* co-culture

Biomass ratio	Biomass A (g/L)	Biomass B (g/L)	Total biomass (g/L)	Abundance	Product concentration (mmol/L)
0.9,0.1	0.67	0.03	0.70	0.96,0.04	0.01
0.8,0.2	0.63	0.06	0.69	0.91,0.09	0.02
0.7,0.3	0.59	0.09	0.68	0.87,0.13	0.03
0.6,0.4	0.54	0.12	0.66	0.81,0.19	0.03
0.5,0.5	0.48	0.16	0.64	0.75,0.25	0.04
0.4,0.6	0.39	0.19	0.58	0.67,0.33	0.06
0.3,0.7	0.33	0.23	0.56	0.59,0.41	0.07
0.2,0.8	0.23	0.27	0.50	0.47,0.53	0.08
0.1,0.9	0.14	0.32	0.46	0.31,0.69	0.09

**Table 6:** Effect of inoculum ratio on the productivity of 1,3-PDO in *S. oneidensis - K. pneumoniae* co-culture

Biomass ratio	Biomass A (g/L)	Biomass B (g/L)	Total biomass (g/L)	Abundance	Product concentration (mmol/L)
0.1,0.9	0.47	0.12	0.59	0.79,0.21	0.04
0.2,0.8	0.40	0.23	0.62	0.64,0.36	0.07
0.3,0.7	0.35	0.33	0.68	0.51,0.49	0.09
0.4,0.6	0.29	0.41	0.7	0.41,0.59	0.11
0.5,0.5	0.23	0.48	0.71	0.32,0.68	0.14
0.6,0.4	0.19	0.57	0.76	0.24,0.76	0.14
0.7,0.3	0.13	0.63	0.76	0.18,0.82	0.18
0.2,0.8	0.09	0.67	0.76	0.11,0.88	0.22
0.9,0.1	0.04	0.75	0.8	0.05,0.95	0.23

### Validation

Though some of the results we obtain corroborate with experimental studies as listed in Table 4, these studies only talk about the biosynthetic capability of the community and not the monoculture. There are very few studies that compare the production capabilities of the monoculture and the community in the same environment. Interestingly, a previous experimental study^29^ attempted to enhance 1,3-propanediol (1,3-PDO) production in *K. pneumoniae* using glycerol as a carbon source under anaerobic conditions. Despite strain engineering efforts, they identified an insufficient supply of reducing power as a major bottleneck for 1,3-PDO synthesis. Their study demonstrated that a *K. pneumoniae*–*Shewanella oneidensis* co-culture outperformed the *K. pneumoniae* monoculture, as *S. oneidensis* functioned as an electron mediator, facilitating improved 1,3-PDO production. Remarkably, *S. oneidensis* proved more effective than both physiological (riboflavin) and non-physiological (neutral red and methyl viologen) electron mediators. Consistent with these findings, our analysis identifies this co-culture as the highest 1,3-PDO producer among all co-cultures and monocultures examined in this study. To demonstrate the reliability of COSMOS, we simulated the growth of the co-culture and the monoculture in the medium used in the experimental study and compared the results. The first analysis is to identify the optimum glycerol concentration for the monoculture. We found 50 g/L glycerol to be the optimum concentration for 1,3-PDO production, which correlates with the experiment values, as shown in Table 7. Although the algorithm captures production trends, discrepancies in absolute values may arise due to limitations in kinetic parameters, oversimplified Michaelis-Menten kinetics, and the absence of regulatory mechanisms. Additionally, Dynamic FBA (dFBA) assumes quasi-steady-state metabolism at each time step, ignoring transient metabolite pools and dynamic regulation, which can contribute to deviations from experimental data.

**Table 7:** Concentration of 1,3 propanediol in experiments and simulations under varying glycerol concentration

Glycerol concentration (g/L)	Concentration of 1,3 PDO in experiments (g/L)	Concentration of 1,3 PDO in COSMOS (g/L)
40	11.3	1.76
50	12.68	1.83
70	12.21	1.58

For the co-culture analysis, the experimental group used a fed-batch reactor under anaerobic conditions where the initial glycerol concentration was 50 g/L. When the glycerol concentration fell below 10 g/L, additional glycerol was introduced to maintain a final concentration of 30 g/L. They found that the co-culture produced 32.01 g/L of 1,3-PDO, which was significantly higher when compared to the monoculture. They also optimised the inoculum ratio and found that a 1:1 ratio of *Klebsiella pneumoniae*: *Shewanella oneidensis* resulted in the highest product concentration, though the exact values are not listed. We did a similar analysis using COSMOS and found that the co-culture was, in fact, able to produce more product in the fed-batch system than the monoculture. However, we found that both the inoculum ratios of 1:1 and 2:1 performed equally well, as shown in Table 8. This might be because we have used the wild-type models of both organisms and lack the strain-specific information that might be required.

**Table 8:** Concentration of 1,3-propanediol with varying inoculum ratio according to simulations

Initial biomass ratio	1,3 Propanediol (g/L)	Abundance
2,1	2.42	0.04, 0.96
1,1	2.20	0.07, 0.93
1,2	0.31	0.1, 0.9
1,3	0.28	0.12, 0.88

The study reported monoculture and co-culture yields of 0.4 g/g and 0.44 g/g, respectively, reflecting a 10% improvement. COSMOS predicted yields of 1.50 g/g for monocultures and 1.33 g/g for co-cultures, indicating a 12.6% increase. Although the absolute values differ, the algorithm effectively captured the trend in performance, identifying optimal substrate concentrations and inoculum ratios. This observation demonstrates the potential of dynamic modelling in bioprocess design, offering a powerful tool for efficiently comparing multiple communities and monocultures to identify the most productive configurations.

## Discussion

Microbial communities often exhibit superior biosynthetic capability compared to monocultures^7,30^. However, their application in bioproduction remains limited to cases where their productivity is already known or where their metabolic diversity is essential, such as when one community member can metabolise a complex carbon source that the others cannot. Studies that directly compare the biosynthetic potential of multiple communities remain scarce^21^, leaving a gap in identifying the most effective microbial systems.

To address this, COSMOS provides a systematic approach to evaluating microbial systems by assessing a wide range of co-cultures alongside their corresponding monocultures. Unlike tools like FLYCOP^20^, which optimise the configuration of a single chosen co-culture, COSMOS systematically evaluates a diverse set of co-cultures alongside their constituent monocultures to identify the optimal microbial system for a given product. The assessment can be tailored to prioritise either productivity or yield, depending on the specific requirements. It also offers granular insights into the system’s performance across a wider range of environmental conditions. COSMOS is particularly valuable when working with low-quality feedstocks, such as agricultural residues and wastewater, which are gaining importance due to global efforts to transition toward second-generation feedstocks for improved food security and sustainability^31^. Given these challenges, optimising the microbial system is just as crucial as optimising the environment to develop efficient and sustainable bioprocesses.

While productivity or yield are often the primary factors in selecting a microbial system, the choice between monocultures and communities depends on additional factors. If a community offers substantially higher productivity or yield, it is the better option for bioproduction. However, when productivity or yield is comparable, monocultures are preferred for their ease of control and simplicity. In cases where operational simplicity takes precedence over maximum output, slightly less productive monocultures can be selected. COSMOS streamlines this decision-making process by assessing the biosynthetic potential of multiple microbial systems under diverse environments.

COSMOS employs parsimonious dFBA^22,23^ to model microbial growth in synthetic consortia, allowing it to capture fluctuating growth rates. While traditional steady-state modelling is simpler to use, it assumes equal growth rates and is better suited to natural communities^32^. Therefore, we use dFBA, which accounts for fluctuating growth and provides promising results even with the lack of organism-specific parameters^33^. This makes COSMOS especially valuable for engineered or synthetic consortia, where kinetic data is often unavailable, and the stability of the co-culture remains uncertain.

By integrating dFBA and FVA, COSMOS minimises redundancy in metabolic predictions, providing more precise insights into productivity^24^. To ensure an unbiased comparison, it evaluates communities and monocultures under identical conditions, benchmarking co-culture productivity against the highest-performing monoculture. This approach prioritises final productivity or yield, making it a more effective tool for selecting optimal bioproduction systems^34^.

To understand the factors that influence bioproduction in co-cultures and monocultures, we tested COSMOS across four environments: aerobic-rich, aerobic-minimal, anaerobic-rich, and anaerobic-minimal media. While overall productivity was highest in the aerobic nutrient-rich conditions, communities outperformed monocultures in anaerobic environments. This trend can be attributed to several factors. Anaerobic environments, characterised by slower and incomplete fermentation, result in more comparable growth rates between species, fostering cooperative interactions, as shown in Table 2. Additionally, the accumulation of intermediate metabolites enhances resource exchange, promoting metabolic cooperation and improved production, as discussed in Table 3 and Section 2.1. Similar observations have been reported in some studies^27^, while others have demonstrated that, in aerobic-rich conditions, organisms prioritise resource utilisation and compete for dominance^35^. These findings further support the notion that microbial communities, in general, can achieve higher production efficiencies in anaerobic environments compared to monocultures.

Interestingly, there were exceptions where communities achieved maximum productivity in aerobic environments, while certain monocultures outperformed communities in anaerobic conditions. This highlights the importance of systematically evaluating both communities and monocultures before selecting the optimal microbial system for a specific product. COSMOS enables us to maximise productivity in the chosen fermentation medium and provides insights into the role of mutualistic interactions in co-culture design. By leveraging these interactions, it aids in optimising both productivity and stability for biomanufacturing applications.

We also examined the effect of carbon sources on these systems. Certain carbon sources, like lactose and sucrose, enable better productivity in the community, while glucose and fructose boost monoculture performance in most environments. Several factors may influence this phenomenon. Most microorganisms are naturally adapted to utilising simple carbon sources like glucose and fructose, which are readily absorbed, leading to resource competition and reduced cooperation in co-cultures. However, in the aerobic-rich environment, this phenomenon is reversed, likely because the abundance of resources enables both organisms to access sufficient glucose, supporting efficient growth and production within the community^36^.

In contrast, the metabolism of alternative carbon sources often involves distinct and less efficient pathways, significantly slowing down the growth of both organisms, resulting in more cooperation. Additionally, certain sugars may be converted into organic acids, which can be exchanged between community members, fostering metabolic cooperation^37^. For instance, *P. aeruginosa* cannot directly metabolise maltose and instead depends on its co-culture partner to break down maltose into glucose before utilisation^38^. Although the patterns of carbon source utilisation are not entirely clear, these findings highlight carbon source selection as a crucial factor in optimising and controlling bioprocesses. This analysis provides a framework for selecting or enriching fermentation media with specific carbon sources to enhance microbial performance. Additionally, the algorithm can be readily extended to assess the impact of multiple carbon sources. Furthermore, COSMOS facilitates the optimisation of initial inoculum ratios, offering another lever to enhance productivity.

To validate COSMOS, we applied it to the *Klebsiella pneumoniae—Shewanella oneidensis* co-culture and its corresponding monocultures, effectively capturing the impact of carbon source concentration and inoculum ratio. Consistent with the findings of previous studies^29^, our analysis confirms that the co-culture outperforms monocultures in 1,3-PDO production. This enhancement is attributed to the superior electron-mediating capability of *S. oneidensis*, which alleviates the redox imbalance in *K. pneumoniae*. Since 1,3-PDO biosynthesis requires high reducing power, *K. pneumoniae* accumulates excess H^+^ ions, which *S. oneidensis* subsequently utilises, as shown in Table 3. This demonstrates that COSMOS accurately captures the metabolic interactions driving improved production. Moreover, the observed trends in yield and productivity across different conditions align well with experimental data, reinforcing the reliability of the algorithm. Additionally, several other microbial communities identified in our study have been experimentally validated as stable consortia, with some already demonstrating promising biosynthetic capabilities, as listed in Table 3.

Although the algorithm effectively captures production trends (Tables 6 and 7), discrepancies in absolute values arise due to several factors. DFBA relies on predefined kinetic parameters that may not fully represent in vivo enzyme kinetics, and the use of Michaelis-Menten kinetics may oversimplify nutrient uptake dynamics. Additionally, dFBA assumes quasi-steady-state metabolism at each time step, neglecting transient metabolite accumulation and regulatory mechanisms, which can lead to deviations in the predictions. Moreover, our model employs wild-type genome-scale reconstructions that may lack strain-specific pathways and alternative metabolic routes. These limitations highlight the challenges of predicting absolute production values while reinforcing dFBA’s utility for capturing overall metabolic trends.

While COSMOS provides valuable results using standard kinetic parameters and wild-type GSMMs, its accuracy can be significantly improved by using experimentally-determined kinetic parameters and strain-specific GSMMs^39,40^. It is important to acknowledge that it assumes uniform ${V}_{\max }$ and ${K}_{m}$ values for all metabolites, which may have reduced the sensitivity of growth predictions to these parameters. Incorporating experimentally determined values for at least a subset of metabolites will enhance the accuracy of the algorithm. However, the availability of high-quality, manually curated models for diverse organisms remains a limitation^41^. Improving the accessibility and accuracy of metabolic models will strengthen computational predictions. Additionally, integrating multi-omic data—such as transcriptomic and proteomic information—into GSMMs could further refine the algorithm’s performance, paving the way for more precise bioprocess optimisation^42^. Although more complex, kinetic modelling that accounts for enzyme-level regulation and experimentally derived parameters could further improve accuracy, making it particularly useful for fine-tuning selected microbial communities. Moreover, to investigate the long-term adaptation and evolution of synthetic consortia, community evolution can be modelled using approaches similar to COMETS^43^ or EvolveX^44^.

Although our study primarily focuses on two-member communities, COSMOS can be readily extended to larger consortia. The addition of a third member could significantly alter community dynamics and potentially enhance productivity. However, the combinatorial explosion in the number of three-member communities (^9^C_3_) makes exhaustive analysis computationally challenging. We anticipate that future advancements in dynamic modelling and computational architecture will enable more efficient design and evaluation of higher-order communities. However, alternative approaches^45^ could be explored to predict the behaviour of complex communities based on simpler ones, further streamlining community design.

Notably, the insights from this study have direct relevance to fields like biofuel production, wastewater treatment, and pharmaceutical biosynthesis^46^. Moreover, by optimising microbial systems for nutrient-limited and waste-derived feedstocks, our approach aligns with the UN Sustainable Development Goals (SDGs), particularly SDG 9 (Industry, Innovation, and Infrastructure) and SDG 12 (Responsible Consumption and Production)^46^. In summary, COSMOS provides a robust computational framework to evaluate and compare the productivity of monocultures and microbial communities, addressing the challenges of experimental testing. It offers insights into how environmental factors, carbon sources, and inoculum ratios affect performance, helping researchers make well-informed decisions about which microbial system to use. Whether the goal is to work with nutrient-dense or nutrient-limited media, our approach ensures the selection of the most effective system, enhancing productivity, process efficiency, and community stability.

As the bioeconomy shifts toward sustainable resource utilisation, leveraging agricultural residues and wastewater for microbial bioprocessing is becoming increasingly critical. While nutrient-rich media remain standard for high-value fermentation, optimising processes in nutrient-poor conditions is essential for achieving carbon-neutral goals. Tools like COSMOS play a pivotal role in this transition by balancing productivity with ease of control. Ultimately, this approach supports the rational design of microbial systems, driving advancements in synthetic biology, sustainable biomanufacturing, and the transition to circular bio-economies.

## Methods

### Flux Balance Analysis (FBA)

Constraint-based modelling provides a powerful framework for analysing metabolic networks by leveraging physicochemical and biological constraints to predict cellular behaviour. The metabolic network of the organism is represented as a stoichiometric matrix $A$ of size $m\times n$ where $m$ is the number of metabolites and $n$ is the number of reactions. The entries in each column of $A$ represent the stoichiometric coefficients of the metabolites involved in a given reaction, defining the mass balance constraints of the metabolic network.

A key approach in constraint-based modelling is FBA, which formulates a linear programming (LP) problem to determine the optimal flux distribution that maximises a predefined cellular objective. The linear programming (LP) problem is denoted by

7 $${\max }_{v}{c}^{T}v\,$$

subject to

8 $$A.v=0$$

while

9 $${v}_{l}\le v\le {v}_{u}$$

where $c$ is a vector of weights denoting the contribution of each reaction to the objective function, ${v}\epsilon \,{R}^{n}$ is the vector of metabolic fluxes, ${v}_{l}$ and ${v}_{u}$ are the lower and upper bounds, respectively.

The steady-state assumption ($A.v=0$) ensures that the intracellular metabolite concentrations remain constant, enforcing mass conservation across reactions. Additionally, thermodynamic and enzymatic constraints, often implemented as flux bounds (${v}_{l}\,,\,{v}_{u}$), restrict infeasible reaction directions and regulate uptake and secretion rates based on experimental data.

### Dynamic FBA

**Fig. 6:**
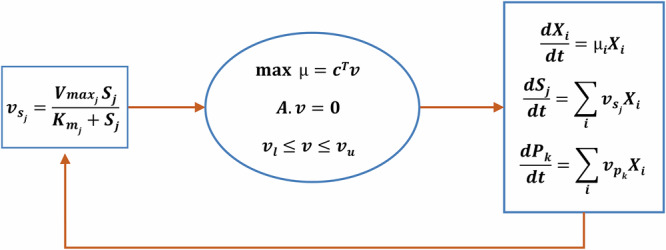
Dynamic flux balance analysis model for a microbial community. The substrate flux ${v}_{{s}_{j}}$ for metabolite $j$ is determined using substrate concentration$\,{S}_{j}$ kinetic parameters ${V}_{\max }$, and ${K}_{m}$ at each timestep. The substrate fluxes, together with the lower and upper bounds, ${v}_{l}$ and ${v}_{u}$ are used to solve the FBA problem. The FBA problem comprises the stoichiometric matrix $A$ and the vector of weights $c$, which represents the contribution of each reaction to the objective function, typically the maximisation of the growth rate, $\mu .$ This optimisation is performed iteratively at each timestep ${dt}$ for each species $i$ using the current concentrations of biomass ${X}_{i}$, substrate flux ${v}_{{s}_{j}}$, and product flux ${v}_{{p}_{k}}$ for product $k$.

dFBA is performed using the static optimisation approach, where the entire batch time is divided into intervals, and the LP is solved at each time interval as a standard FBA problem. While steady-state modelling is well-suited for natural consortia that inherently grow together, dFBA is more appropriate for artificial consortia. Traditional FBA assumes that intracellular fluxes remain at a steady state at each time point, but in dynamic systems, both intracellular and extracellular variables vary over time. Therefore, dFBA extends FBA by incorporating temporal changes, enabling a more accurate representation of community dynamics. The growth rate (μ) and intracellular fluxes ($v$), including product secretion rates (${v}_{p}$) at any given time point, are determined by solving a standard FBA problem at the biomass concentration $X$. Instead of using fixed substrate uptake rates as in the case of classic FBA, we utilise extracellular concentrations of substrates (*S*) and products (*P*) to calculate dynamic substrate uptake rates (${v}_{s}$) based on specific kinetic parameters, as shown in Fig. 6. These rates reflect the maximum uptake capabilities at each time point and are applied as constraints in the calculations.

Once the biomass values are obtained through the dFBA formulation (as shown in Fig. 6), the relative abundance of each species is calculated using the formula:

10 $${{Abundance}}_{i}={X}_{i}/\sum _{i}{X}_{i}$$

This approach is similar to the methodology used in SteadyCom^32^, where species abundances are determined based on biomass concentrations.

#### Calculation of substrate uptake rate

The substrate uptake limit is limited by two factors—the amount of nutrient available in the medium for each species $i$ and the transport kinetics for the substrate. We have used a similar approach as previous studies^47–49^ to calculate the substrate uptake rate. The nutrient concentration in the medium is denoted by ${S}_{j}(t)$, where the maximum amount of nutrient $j$ that species $i$ can import per gram biomass per hour is ${S}_{j,{conc}}$

11 $${S}_{j,{conc}}=\frac{{S}_{j}\left(t\right)}{{X}_{i}\left(t\right)* \Delta t}$$

The second limitation is transport, where the cell’s transport mechanism may not be able to import all the available nutrients. Nutrient transport often follows Michaelis–Menten kinetics^50,51^, and we therefore use the kinetic parameters ${V}_{\max }$ and ${K}_{m}$ to calculate the transport limit ${S}_{j,{trans}}$

12 $${S}_{j,{trans}}=\frac{{{V}_{\max }* S}_{j}\left(t\right)}{{K}_{m}+{S}_{j}\left(t\right)}$$

The substrate uptake rate ${v}_{s}$ is the minimum of these two limits and is given by

13 $${v}_{s}=\min \left[\frac{{S}_{j}\left(t\right)}{\left({X}_{i}\left(t\right)* \Delta t\right)},\frac{{{V}_{\max }* S}_{j}\left(t\right)}{{K}_{m}+{S}_{j}\left(t\right)}\right]$$

This substrate uptake rate is used as an additional constraint for the parsimonious FBA to compute the growth rate ${\mu }_{i}$ of each species $i$ and the medium concentration for each substrate is updated.

#### Flux Variability Analysis (FVA)

To evaluate the production of each product under study, we performed an FVA at each time step. The maximum flux through the exchange reaction for $k$ products ${v}_{{p}_{k}}$ is evaluated as follows

14 $${\max }_{v}{v}_{{p}_{k}}$$

subject to

15 $$A.v=0$$

while

16 $${v}_{l}\le v\le {v}_{u}$$

The product concentration ${P}_{k,i}$ for product $k$ in species $i$ is given by

17 $${P}_{k,i}={v}_{{p}_{k}}* {X}_{i}\left(t\right)* \Delta t$$

FBA provides a unique solution for the objective function; however, fluxes through other reactions can have multiple feasible solutions. To avoid selecting arbitrary redundant solutions, FVA is performed at each time point to determine the range of possible flux values. Comparing the upper bounds of these flux ranges allows for a more accurate assessment of whether a true increase in performance is observed when comparing different systems.

### Comparative analysis

High-quality GSMMs of more than 30 organisms were obtained from the BiGG models database^52^ and the BioModels database^53^. These models were filtered based on criteria such as pathogenicity, prior evidence of bioproduction, model quality, and annotation compatibility^54^. We chose ten organisms for our final analysis, and though some of these are pathogenic (Supplementary Table 1a), bioproduction has been successfully demonstrated in all of them^55–60^. Supplementary Table 1b provides a detailed list of organisms that were considered but excluded from the study due to factors such as poor model quality and other limitations. These models were used to form pairwise communities with a universal compartment shared by the organisms, representing the external medium. The biomass synthesis reactions are defined as the objective function of each organism, allowing them to optimise their growth. Organisms can freely utilise any available metabolites in the extracellular medium, either supplied through initial medium constraints or secreted by the co-existing organisms. The uptake kinetic parameters ${{V}}_{\max }$ and ${K}_{m}$ were assumed to be constant (${V}_{\max }$ = 20 mmol/gDW/h and ${K}_{m}$ = 0.05 mmol) for all metabolites, as the parameters for all the organisms were not readily available.

Many previous studies^47,61^ have assumed a similar ${V}_{\max }$ of 20 mmol/gDW/h for all nutrients, based on the reported distribution of values for nutrient transport. This is comparable to the ${{V}}_{\max }$ of 26 mmol/gDW/h reported for glucose transport in *E. coli*^62^. Similarly, we assume ${K}_{m}$ = 0.05 mmol for all nutrient transport, which falls within the broad range of values documented in the BRENDA enzyme database^47,63,64^. Another study^65^ has demonstrated that variations in ${{V}}_{\max }$ have little to no impact on microbial growth. To further assess the influence of ${{V}}_{\max }$ and ${K}_{m}$ on microbial growth, we performed a sensitivity analysis using Latin Hypercube Sampling (LHS) to generate 10 parameter sets within the ranges ${{V}}_{\max }\,$= 1–50 mmol/gDW/h and ${K}_{m}\,$= 0.01–1 mmol. Multiple regression analysis revealed that ${{V}}_{\max }$ had a coefficient of 0, while ${K}_{m}$ had a coefficient of 0.17, with an *R*^2^ value of 0.02, indicating minimal influence of these parameters on community growth. The limited effect observed may be attributed to the influence of additional factors governing microbial growth, such as substrate availability, oxygen concentration, and co-factor limitations, which can serve as rate-limiting steps or physiological constraints. This observation aligns with previous studies that have reported similar findings^65^.

**Fig. 7:**
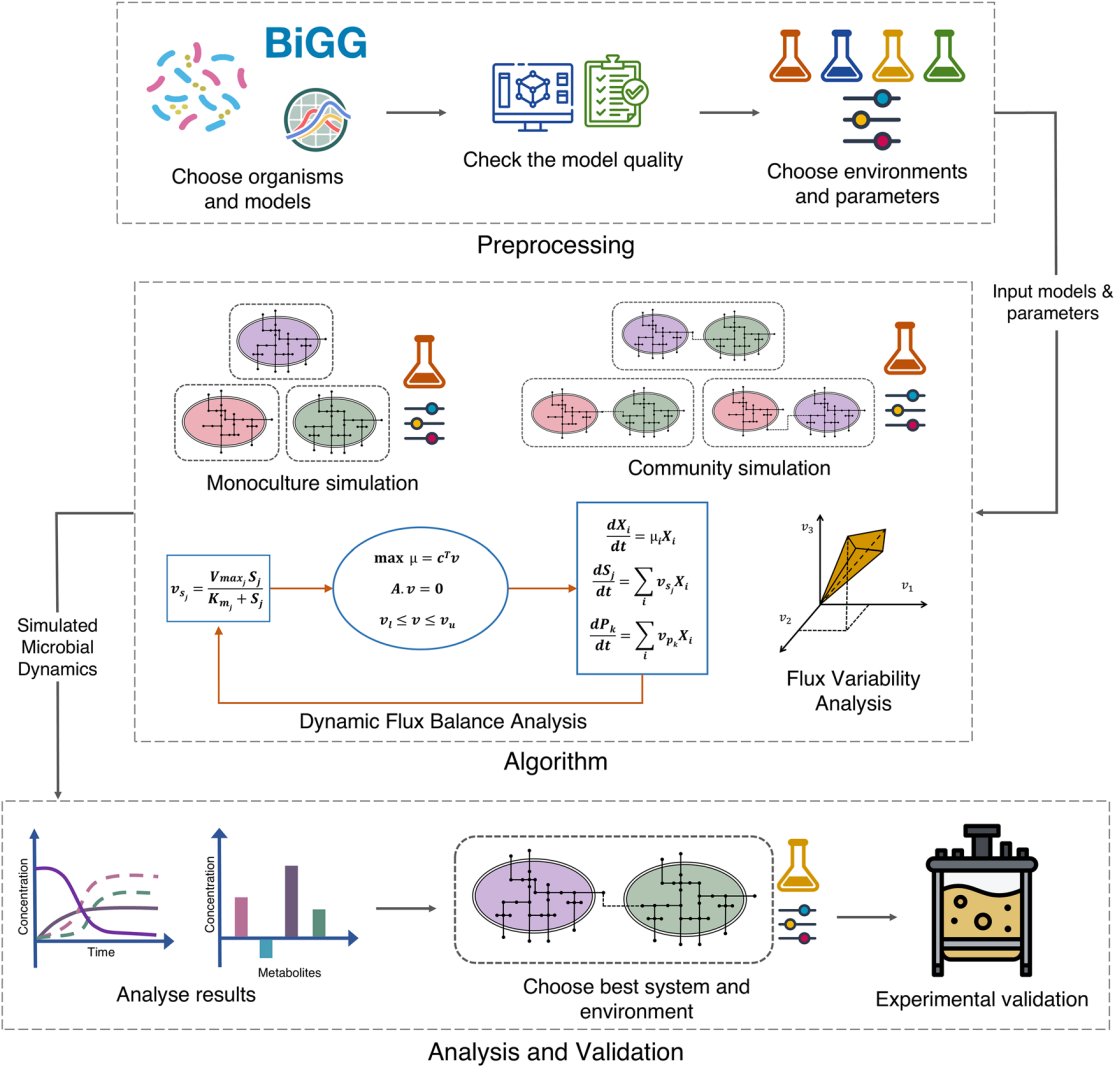
Overview of the COSMOS workflow. The workflow begins with Preprocessing, where the organisms and models are selected, model quality is verified, and parameters are defined. These inputs are then passed to the Algorithm, where dynamic Flux Balance Analysis (dFBA) and Flux Variability Analysis (FVA) are used to simulate microbial growth and metabolite exchange. Finally, in Analysis and Validation, the results are examined to identify optimal microbial systems, which can be experimentally validated when necessary. (Icons: Flaticon.com).

The pairwise community and the monoculture growth rates were simulated in the same external medium, and the product concentrations in both cases were compared to find the optimal system under each scenario and target product, as shown in Fig. 7. To obtain a robust, numerically stable solution for each system, the final biomass, substrate, and product concentrations were averaged over the last five feasible solutions/time points. It is important to note that no collective community biomass objective is being optimised. Instead, each organism independently maximises its own growth, leading to competition within the community. If one organism outgrows the other entirely, the community is considered non-viable. To ensure viability, a community must meet two criteria: a minimum abundance threshold of 0.1 and at least a 10% increase in biomass, consistent with standard thresholds in the literature^66^. These criteria ensured the inclusion of communities where both organisms exhibited substantial growth.

To investigate the impact of medium composition, we designed two types of media: a ‘minimal medium’ and a ‘rich medium.’ The ‘rich medium’ contains over 60 metabolites commonly found in growth media^67^. In contrast, the minimal medium consists of around 40 essential metabolites required for the growth of all the individual organisms under study. A complete list of organisms and media components is provided in Supplementary Tables 1–3.

The initial concentration of each medium component was set to 10 mmol/L to ensure consistency and align with values used in previous studies^68^. To assess the impact of oxygen availability, we simulated community growth under both aerobic (10 mmol/L oxygen) and anaerobic conditions. Obligate anaerobes were excluded from aerobic simulations, and obligate aerobes were excluded from anaerobic simulations (Supplementary Table 1). This resulted in nine aerobic and nine anaerobic organisms, forming ^9^C_2_ pairwise communities, each analysed across four distinct environments: aerobic-rich, aerobic-minimal, anaerobic-rich, and anaerobic-minimal. An initial biomass of 0.01 g/L was chosen for both the organisms of the co-culture to ensure sufficient growth. All simulations were run for a bacterial growth period of 12 h, by which the carbon source was fully consumed, and the organisms reached the stationary phase.

All simulations were performed using MATLAB R2018a, COBRA Toolbox v3.0, and IBM CPLEX solver v12.8. The computation time for a single co-culture and its corresponding monocultures, evaluated for a single product, averages 10 min and scales proportionally. For the analysis of ^9^C_2_ co-cultures (Supplementary Table 1) across 25 products (Supplementary Table 4) in this study, the total computation time is 6.47 h.

### Statistical analysis

The distribution of the data was assessed using the Shapiro–Wilk test, which indicated a non-normal distribution. Consequently, we applied non-parametric statistical methods for hypothesis testing. To model relationships between variables, we implemented Generalised Additive Model (GAM) regression in R (mgcv package). GAMs were selected over simpler models, such as linear regression, due to their ability to flexibly capture nonlinear relationships without requiring a predefined functional form. This was particularly important in our study, as preliminary analyses showed that linear models failed to adequately describe the observed patterns, necessitating a more adaptive approach.

Given the multiple factors influencing the system (e.g., environment, interaction type, carbon source, etc.), we first used GAMs to model their effects and extract residuals, ensuring that statistical comparisons focused solely on the underlying relationships of interest. For group-wise comparisons, we used the Kruskal–Wallis test, a non-parametric alternative to ANOVA. If a statistically significant difference was detected (${p} < \,0.05$), we conducted post-hoc pairwise comparisons using the Dunn test with Benjamini–Hochberg correction to adjust for multiple comparisons. All statistical analyses were performed in *R*^69^ (version 4.4.2; http://www.r-project.org) using the mgcv^70^ (version 1.9–1) and stats^69^ (version 4.4.2) packages.

## Supplementary information

Supplementary information

Supplementary Data 1

Supplementary Data 2

Supplementary Data 3

Supplementary Data 4

Supplementary Data 5

Supplementary Data 6

Supplementary Data 7

Supplementary Data 8

## Data Availability

All models and analysis scripts used in this study are openly available at https://github.com/RamanLab/COSMOS.
