# Supplementary material for: COmmunity and Single Microbe Optimisation System (COSMOS)
Source: NPJ Syst Biol Appl. 2025 May 21;11:51. doi: 10.1038/s41540-025-00534-w (PMC12095823; doi:10.1038/s41540-025-00534-w)
Supplement: Supplementary file 1 — Supplementary information [file 41540_2025_534_MOESM1_ESM.pdf]

Contents

- Description of Supplementary Data
- Supplementary Figures
    - Supplementary Figure 1 – Effect of carbon source on the biosynthetic capability of different products in communities vs monocultures.
- Supplementary Tables
    - Supplementary Table 1a – List of organisms used in this study
    - Supplementary Table 1b – List of organisms evaluated for the study
    - Supplementary Table 2 – Minimal Medium composition
    - Supplementary Table 3 – Rich Medium composition
    - Supplementary Table 4 – List of products
    - Supplementary Table 5 – Best microbial system under different environments across four products
    - Supplementary Table 6 – Best microbial system under different environments across all products
    - Supplementary Table 7 – Growth rates of the organisms in the aerobic-rich medium

Description of Supplementary Data

Supplementary Data 1 – Aerobic - Rich environment analysis

Supplementary Data 2 – Aerobic - Minimal environment analysis

Supplementary Data 3 – Anaerobic - Rich environment analysis

Supplementary Data 4 – Anaerobic - Minimal environment analysis

Supplementary Data 5 – Statistical analysis of the effect of environment on productivity ratio

Supplementary Data 6 – Statistical analysis of the effect of interaction on productivity ratio

Supplementary Data 7 – Statistical analysis of the effect of carbon source on productivity ratio

Supplementary Data 8 – Statistical analysis of the effect of community on productivity ratio

Supplementary Figures

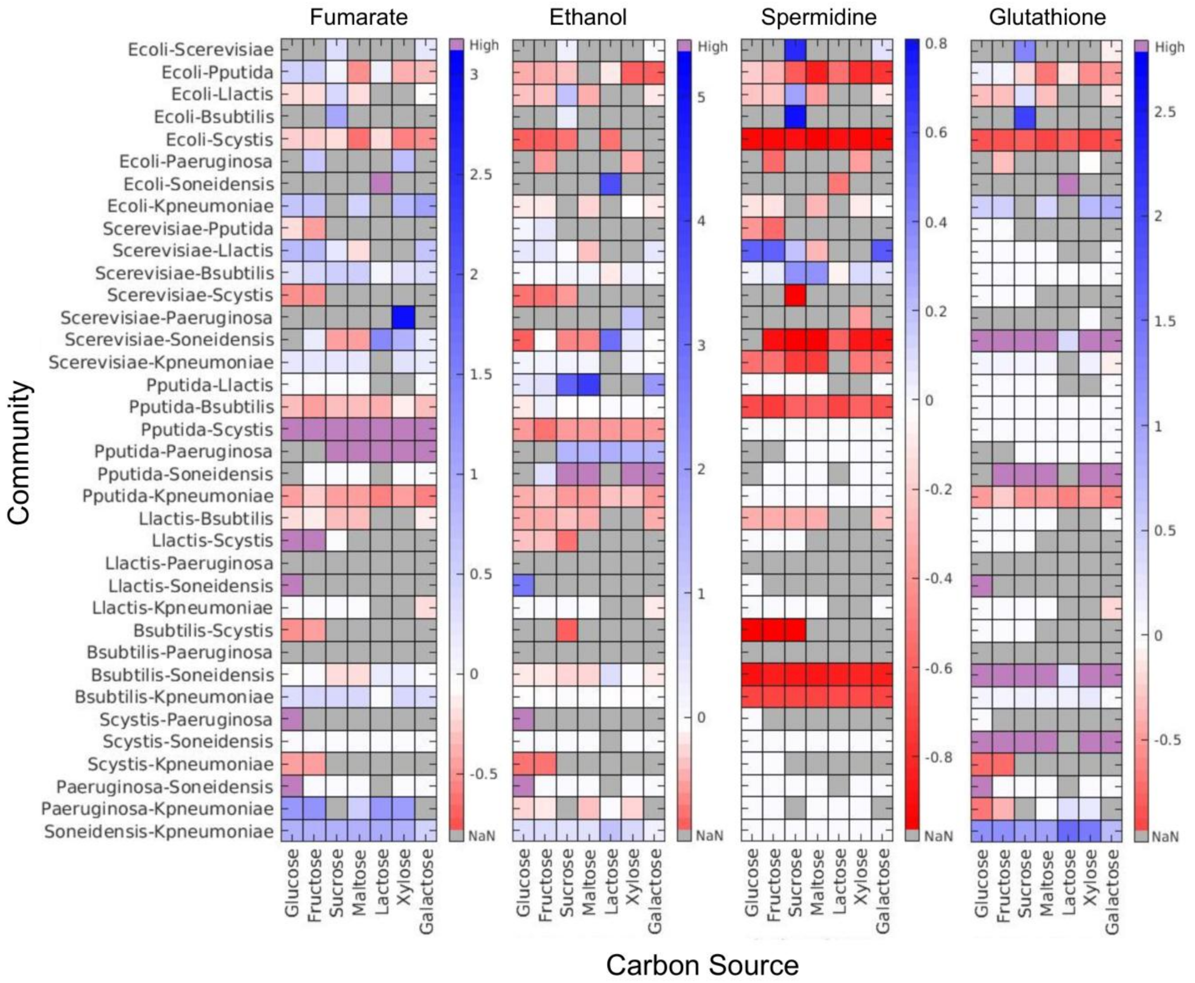

Suppl. Fig 1. Effect of carbon source on the biosynthetic capability of different products in communities vs monocultures. This figure illustrates the productivity ratio of specific products across different communities under varying carbon sources in an aerobic-rich environment. Positive values (blue) indicate an increase in the productivity of a given product in the community compared to the monocultures, while negative values (red) reflect a decline in productivity. If the product is only produced by the community and not by the monocultures, the value is shown in purple and labelled as 'High.' Conversely, when the community fails to grow on a specific carbon source, the value is represented in grey and marked as 'NaN.'

Supplementary Tables

Supplementary Table 1a. List of organisms used in this study

S.No.	Organism	Aerobic/Anaerobic	Pathogenicity	Evidence of Bioproduction
1	*Escherichia coli*	Both	No	[1]
2	*Saccharomyces cerevisiae*	Both	No	[2]
3	*Pseudomonas putida*	Obligate aerobe	No	[3]
4	*Lactococcus lactis*	Both	No	[4]
5	*Bacillus subtilis*	Both	No	[5]
6	*Synechocystis spp.*	Both	No	[6]
7	*Pseudomonas aeruginosa*	Both	Opportunistic pathogen	[7]
.8	*Shewenella oneidensis*	Both	Opportunistic pathogen	[8,9]
9	*Klebsiella pneumoniae*	Both	Opportunistic pathogen	[10,11]
10	*Clostridium ljungdahlii*	Obligate anaerobe	No	[12]

Supplementary Table 1b. List of organisms evaluated for the study

S.No.	Organism	Aerobic/ Anaerobic	Pathogenicity	Reason for exclusion
1	*Methanosarcina barkeri*	Obligate anaerobe	No	Methylotrophic
2	*Thermotoga maritima*	Obligate anaerobe	No	Extremophile
3	*Clostridium beijerinckii*	Obligate anaerobe	No	Poor model quality
4	*Zymomonas mobilis*	Both	No	Poor model quality
5	*Rhodobacter sphaeroides*	Both	No	Poor model quality
6	*Methylobacterium extorquens*	Both	No	Poor model quality
7	*Acinetobacter baylyi*	Obligate aerobe	No	Poor model quality
8	*Yarrowia lipolytica*	Obligate aerobe	No	Poor model quality
9	*Bifidobacterium adolescentis*	Obligate anaerobe	No	Poor model quality
10	*Bacteroides thetaiotaomicron*	Obligate anaerobe	No	No evidence of bioproduction
11	*Faecalibacterium prausnitzii*	Obligate anaerobe	No	No evidence of bioproduction

12	*Helicobacter pylori*	Both	Pathogen	No evidence of bioproduction
13	*Synechococcus elongatus*	Both	No	Poor model quality
14	*Chlamydomonas reinhardtii*	Both	No	Poor model quality
15	*Acinetobacter baumanii*	Obligate aerobe	Opportunistic pathogen	No evidence of bioproduction
16	*Porphyromonas gingivalis*	Obligate anaerobe	Opportunistic pathogen	No evidence of bioproduction
17	*Shigella boydii*	Both	Pathogen	No evidence of bioproduction
18	*Salmonella enterica*	Both	Pathogen	No evidence of bioproduction
19	*Aspergillus nidulans*	Obligate aerobe	Opportunistic pathogen	No evidence of bioproduction – except enzymes
20	*Burkholderia cenocepacia*	Obligate aerobe	Opportunistic pathogen	No evidence of bioproduction
21	*Clostridium glutamicum*	Both	No	Poor model quality
22	*Methanococcus maripuladis*	Obligate anaerobe	No	Methylotrophic

Supplementary Table 2. Minimal Medium composition

**Minimal medium**	
Glucose	Oxygen
4 Aminobenzoate	Phosphate
Biotin	Pantothenate
Calcium	Riboflavin
Cobalamin	Sulphate
Chlorine	Zinc
Cobalt	Selenium
Copper(II)	Thiamine
Iron(II)	Pyridoxal
Iron(III)	Hydrogen sulphide
Folate	Uracil
Hydrogen	L-alanine
Water	L-glutamate
Potassium	L-leucine
Magnesium	L-threonine
Manganese	L-valine
Molybdenum	L-isoleucine
Sodium	L-arginine
Nicotinamide	L-serine
Ammonium	Nicotinate
Nickel	Sulphite
Nitrate	

Supplementary Table 3. Rich Medium composition

Rich Medium	
Glucose	L-alanine
4 Aminobenzoate	L-glutamate
Biotin	L-leucine
Calcium	L-threonine
Cobalamin	L-valine
Chlorine	L-isoleucine
Cobalt	L-arginine
Copper(II)	L-serine
Iron(II)	Nicotinate
Iron(III)	Sulphite
Folate	L-aspartate
Hydrogen	L-asparagine
Water	L-cysteine
Potassium	L-glutamine
Magnesium	L-glycine
Manganese	L-histidine
Molybdenum	L-lysine
Sodium	L-methionine
Nicotinamide	L-phenylalanine
Ammonium	L-proline
Nickel	L-tryptophan
Nitrate	L-tyrosine
Oxygen	Adenosylcobalamin
Phosphate	Guanine
Pantothenate	Orotate
Riboflavin	Xanthine
Sulphate	Lead
Zinc	Protoheme
Selenium	Pimelate
Thiamine	Choline
Pyridoxal	Thymidine
Hydrogen sulphide	Carbon Dioxide
Uracil	

Supplementary Table 4. List of products

#	Product	Classification
1	Succinate	Organic acid
2	Pyruvate	
3	Formate	
4	L-Lactate	
5	D-Lactate	
6	Acetate	
7	Fumarate	
8	Gluconate	
9	Propionate	Carboxylic acid
10	Adipic acid	
11	Sorbitol	Sugar alcohol
12	Xylitol	
13	Ethanol	Alcohol
14	Methanol	
15	Butanol	
16	Propane-1,2-diol	Diol
17	Propane-1,3-diol	
18	2,3 Butanediol	
19	Glycerol	Triol
20	Hydrogen	Gas
21	Butyrate	Scfa
22	Spermidine	Polyamine
23	Putrescine	
24	Catechol	Phenol
25	Glutathione	Tripeptide

Supplementary Table 5. Best microbial system under different environments across four products

Aerobic Rich Environment								
#	Fumarate		Ethanol		Spermidine		Glutathione	
	Microbial system	Productivity (mmol/L/hr)	Microbial system	Productivity (mmol/L/hr)	Microbial system	Productivity (mmol/L/hr)	Microbial system	Productivity (mmol/L/hr)
1	*P. aeruginosa - K. pneumoniae*	1.27	*S. oneidensis - K. pneumoniae*	2.04	*E. coli*	0.07	*S. oneidensis - K. pneumoniae*	0.26
2	*E. coli - K. pneumoniae*	1.21	*S. cerevisiae - K. pneumoniae*	1.42	*B. subtilis*	0.05	*E. coli - K. pneumoniae*	0.25
3	*S. oneidensis - K. pneumoniae*	1.10	*K. pneumoniae*	1.29	*S. cerevisiae - L. lactis*	0.02	*E. coli - P. putida*	0.19
4	*E. coli - P. putida*	1.07	*E. coli*	1.10	*S. cerevisiae*	0.03	*E. coli*	0.17
5	*B. subtilis - K. pneumoniae*	0.88	*P. aeruginosa - S. oneidensis*	1.04		0.02	*B. subtilis - K. pneumoniae*	0.13
**Aerobic Minimal Environment**								
#	Fumarate		Ethanol		Spermidine		Glutathione	
	Microbial system	Productivity (mmol/L/hr)	Microbial system	Productivity (mmol/L/hr)	Microbial system	Productivity (mmol/L/hr)	Microbial system	Productivity (mmol/L/hr)
1	*P. aeruginosa - K. pneumoniae*	0.58	*S. oneidensis - K. pneumoniae*	1.12	*S. cerevisiae - B. subtilis*	0.029	*E. coli - K. pneumoniae*	0.06
2	*E. coli - K. pneumoniae*	0.50	*S. cerevisiae - P. aeruginosa*	0.73	*E. coli - P. aeruginosa*	0.026	*E. coli - P. putida*	0.043

3	*S. cerevisiae - P. aeruginosa*	0.47	*P. putida - S. oneidensis*	0.73	*E. coli - S. cerevisiae*	0.024	*S. oneidensis - K. pneumoniae*	0.042
4	*E. coli - P. aeruginosa*	0.46	*P. aeruginosa - K. pneumoniae*	0.69	*E. coli - P. putida*	0.022	*K. pneumoniae*	0.038
5	*Synechocystis - P. aeruginosa*	0.38	*E. coli - S. cerevisiae*	0.65	*E. coli - L. lactis*	0.019	*E. coli - P. aeruginosa*	0.030
**Anaerobic Rich Environment**								
**#**	**Fumarate**		**Ethanol**		**Spermidine**		**Glutathione**	
	**Microbial system**	**Productivity (mmol/L/hr)**	**Microbial system**	**Productivity (mmol/L/hr)**	**Microbial system**	**Productivity (mmol/L/hr)**	**Microbial system**	**Productivity (mmol/L/hr)**
1	*E. coli - S. oneidensis*	1.03	*E. coli - S. oneidensis*	1.37	*E. coli - S. oneidensis*	0.07	*E. coli - S. oneidensis*	0.26
2	*E. coli - K. pneumoniae*	0.89	*C.* ljungdahlii - *K. pneumoniae*	0.97	*E. coli - K. pneumoniae*	0.06	*E. coli - K. pneumoniae*	0.16
3	*P. aeruginosa - S. oneidensis*	0.33	*S. oneidensis - K. pneumoniae*	0.79	*C. ljungdahlii - B. subtilis*	0.02	*S. oneidensis - K. pneumoniae*	0.10
4	*S. oneidensis - K. pneumoniae*	0.33	*C. ljungdahlii - B. subtilis*	0.76	*E. coli*	0.02	*P. aeruginosa - S. oneidensis*	0.07
5	*B. subtilis - K. pneumoniae*	0.29	*P. aeruginosa - S. oneidensis*	0.68	*B. subtilis*	0.01	*E. coli*	0.05
**Anaerobic Minimal Environment**								
**#**	**Fumarate**		**Ethanol**		**Spermidine**		**Glutathione**	
	**Microbial system**	**Productivity (mmol/L/hr)**	**Microbial system**	**Productivity (mmol/L/hr)**	**Microbial system**	**Productivity (mmol/L/hr)**	**Microbial system**	**Productivity (mmol/L/hr)**

1	*P. aeruginosa - K. pneumoniae*	0.34	*P. aeruginosa - S. oneidensis*	0.54	*E. coli - P. aeruginosa*	0.024	*E. coli - P. aeruginosa*	0.04
2	*E. coli - P. aeruginosa*	0.31	*P. aeruginosa - K. pneumoniae*	0.45	*E. coli - C. ljungdahlii*	0.0049	*P. aeruginosa - K. pneumoniae*	0.02
3	*P. aeruginosa - S. oneidensis*	0.20	*E. coli - C. ljungdahlii*	0.38	*C. ljungdahlii - B. subtilis*	0.0047	*S. oneidensis - K. pneumoniae*	0.02
4	*B. subtilis - K. pneumoniae*	0.10	*E. coli - P. aeruginosa*	0.36	*B. subtilis*	0.0041	*P. aeruginosa - S. oneidensis*	0.01
5	*S. oneidensis - K. pneumoniae*	0.09	*E. coli - S. oneidensis*	0.34	*E. coli*	0.0027	*B. subtilis - K. pneumoniae*	0.01

Supplementary Table 6. Best microbial system under different environments across all products

#	Aerobic-rich		Aerobic-minimal		Anaerobic-rich		Anaerobic-minimal	
	**Microbial system**	**No. of Products**	**Microbial system**	**No. of Products**	**Microbial system**	**No. of Products**	**Microbial system**	**No. of Products**
1	*E. coli*	16	*E. coli*	15	*E. coli*	16	*E. coli - P. aeruginosa*	16
2	*K. pneumoniae*	14	*K. pneumoniae*	14	*E. coli - K. pneumoniae*	16	*E. coli*	15
3	*S. cerevisiae*	14	*S. cerevisiae - L. lactis*	14	*E. coli - S. oneidensis*	16	*B. subtilis - K. pneumoniae*	14
4	*S. cerevisiae - L. lactis*	14	*E. coli - P. aeruginosa*	13	*K. pneumoniae*	13	*E. coli - C. ljungdahlii*	14
5	*S. cerevisiae - K. pneumoniae*	13	*E. coli - K. pneumoniae*	12	*S. oneidensis - K. pneumoniae*	13	*P. aeruginosa - K. pneumoniae*	14
6	*S. oneidensis - K. pneumoniae*	13	*S. cerevisiae*	12	*B. subtilis*	11	*S. oneidensis - K. pneumoniae*	13
7	*B. subtilis*	12	*S. cerevisiae - P. aeruginosa*	12	*P. aeruginosa - S. oneidensis*	11	*K. pneumoniae*	12
8	*B. subtilis - K. pneumoniae*	11	*S. oneidensis - K. pneumoniae*	12	*B. subtilis - K. pneumoniae*	9	*P. aeruginosa - S. oneidensis*	11
9	*E. coli - K. pneumoniae*	11	*B. subtilis*	11	*C. ljungdahlii - B. subtilis*	8	*C. ljungdahlii - B. subtilis*	10
10	*P. aeruginosa - S. oneidensis*	11	*E. coli - P. putida*	10	*L. lactis - S. oneidensis*	8	*E. coli - S. oneidensis*	10

Supplementary Table 7. Growth rates of the organisms in the aerobic-rich medium

Organism	Observed growth rate (g/L/hr)
Escherichia coli	0.49
Saccharomyces cerevisiae	0.28
Pseudomonas putida	0.28
Lactococcus lactis	0.09
Bacillus subtilis	0.31
Synechocystis sp. PCC 6803	0.07
Pseudomonas aeruginosa	0.11
Shewanella oneidensis	0.03
Klebsiella pneumoniae	0.47

References:

1. Gaur, V. K. *et al.* Efficient bioproduction of poly(3-hydroxypropionate) homopolymer using engineered *Escherichia coli* strains. *Bioresource Technology* **397**, 130469 (2024).
2. Wang, Z. *et al.* Synthetic evolution of Saccharomyces cerevisiae for biomanufacturing: Approaches and applications. *mLife* **4**, 1–16 (2025).
3. Mezzina, M. P., Manoli, M. T., Prieto, M. A. & Nikel, P. I. Engineering Native and Synthetic Pathways in Pseudomonas putida for the Production of Tailored Polyhydroxyalkanoates. *Biotechnology Journal* **16**, 2000165 (2021).
4. Aso, Y., Hashimoto, A. & Ohara, H. Engineering Lactococcus lactis for D-Lactic Acid Production from Starch. *Curr Microbiol* **76**, 1186–1192 (2019).
5. Su, Y., Liu, C., Fang, H. & Zhang, D. Bacillus subtilis: a universal cell factory for industry, agriculture, biomaterials and medicine. *Microb Cell Fact* **19**, 173 (2020).
6. Yunus, I. S. *et al.* Improved Bioproduction of 1-Octanol Using Engineered Synechocystis sp. PCC 6803. *ACS Synth. Biol.* **10**, 1417–1428 (2021).

7. Patel, A. T., Akhani, R. C., Patel, M. J., Dedania, S. R. & Patel, D. H. Bioproduction of l-Aspartic Acid and Cinnamic Acid by l-Aspartate Ammonia Lyase from Pseudomonas aeruginosa PAO1. *Appl Biochem Biotechnol* **182**, 792–803 (2017).
8. Ikeda, S. *et al.* Shewanella oneidensis MR-1 as a bacterial platform for electro-biotechnology. *Essays in Biochemistry* **65**, 355–364 (2021).
9. Jeon, J.-M. *et al.* Isobutanol production from an engineered Shewanella oneidensis MR-1. *Bioprocess Biosyst Eng* **38**, 2147–2154 (2015).
10. Mitrea, L. & Vodnar, D. C. Klebsiella pneumoniae—A Useful Pathogenic Strain for Biotechnological Purposes: Diols Biosynthesis under Controlled and Uncontrolled pH Levels. *Pathogens* **8**, 293 (2019).
11. Oh, B.-R. *et al.* Fermentation strategies for 1,3-propanediol production from glycerol using a genetically engineered Klebsiella pneumoniae strain to eliminate by-product formation. *Bioprocess Biosyst Eng* **35**, 159–165 (2012).
12. Acharya, B., Dutta ,Animesh & and Basu, P. Ethanol production by syngas fermentation in a continuous stirred tank bioreactor using Clostridium ljungdahlii. *Biofuels* **10**, 221–237 (2019).
